# Hyper-altruistic behavior vanishes with high stakes

**DOI:** 10.1371/journal.pone.0255668

**Published:** 2021-08-25

**Authors:** Pablo Brañas-Garza, Diego Jorrat, Jaromír Kovářík, María C. López

**Affiliations:** 1 Department of Economics, Universidad Loyola Andalucía, Córdoba, Spain; 2 Loyola Behavioral Lab, Universidad Loyola Andalucía, Córdoba, Spain; 3 Department of Economic Analysis, University of the Basque Country UPV-EHU, Bilbao, Spain; 4 CERGE-EI, Prague, Czech Republic; 5 Faculty of Arts, University of West Bohemia, Pilsen, Czech Republic; Baylor University, UNITED STATES

## Abstract

Using an incentivized experiment with statistical power, this paper explores the role of stakes in charitable giving of lottery prizes, where subjects commit to donate a fraction of the prize before they learn the outcome of the lottery. We study three stake levels: 5€ (*n* = 177), 100€ (*n* = 168), and 1,000€ (*n* = 171). Although the donations increase in absolute terms as the stakes increase, subjects decrease the donated fraction of the pie. However, people still share roughly 20% of 1,000€, an amount as high as the average monthly salary of people at the age of our subjects. The number of people sharing 50% of the pie is remarkably stable across stakes, but donating the the whole pie–the modal behavior in charity-donation experiments–disappears with stakes. Such hyper-altruistic behavior thus seems to be an artifact of the stakes typically employed in economic and psychological experiments. Our findings point out that sharing with others is a prevalent human feature, but stakes are an important determinant of sharing. Policies promoted via prosocial frames (e.g., stressing the effects of mask-wearing or social distancing on others during the Covid-19 pandemic or environmentally-friendly behaviors on future generations) may thus be miscalibrated if they disregard the stakes at play.

## Introduction

Generosity toward others and particularly sharing with those less fortunate are fundamental features of most human societies and religions around the Globe [1–3]. Over 30 years of experimental research across fields has documented that people are fairly generous in the lab and in the field even toward unrelated others. Generosity is traditionally measured via the Dictator Game (DG, henceforth), proposed by [4]. DG is a 2-player game, in which one player, a dictator, is asked to divide a certain amount of money between himself and another–normally unknown and anonymous–recipient who can only accept the division. In general, dictators donate an average of 30% of the money and only a minority keep the entire amount for themselves; when the recipient is a charity, the donations are even higher and more than 20% actually donate the whole pie (see [5, 6], for a meta-study). Although these numbers support the idea of “pro-social” human behavior, the vast majority of research has involved relatively low stakes under *certainty*, where certainty refers to situations, in which subjects know the size of the pie, the amount of money they keep, and the amount of money the recipients receive.

Notwithstanding this, many relevant real-life situations simulated by the employed experimental protocols involve large degree of ambiguity and the stakes at play are considerable. Under *uncertainty*, at least one aspect of the situation is not known with certainty. We term *risk* uncertain settings, in which the probabilities are known, while *ambiguity* refers to situations where the probabilities of different events are unknown [7–9]. There is an agreement in economics that ambiguity differs behaviorally from risk [9–13]. For example, donors to charities are rarely fully aware of how their money will be used and to what extent their goals will be achieved; parents while sharing with/saving for their offspring cannot predict how their “gifts” will affect their children’s life; physicians exert costly effort on their patients even though the result of their work depends on a myriad of aspects out of their control; or the money inverted in the prevention of climate change for future generations has largely unpredictable consequences. Since all these and many other real-life acts of altruism toward others involve considerable degree of ambiguity and large stakes, to what extent can we extrapolate experimentally observed sharing behavior to real-life scenarios [14]? One particular puzzle while giving to charities in experiments is the frequent donation of the whole pie [6]–labeled as *hyper-altruism* throughout this study–that contrasts starkly with reality, where donating the whole income is rather a rarity [15].

This study explores the impact of stakes on donations of lottery prizes to charities. We argue that sharing, say, 5€ with a charity differs both morally and psychologically from sharing 1,000€ and hypothesize that large stakes might undermine altruistic motives.

We follow the literature and employ the DG. To study the role of stakes, we conduct three treatments with respect to the pie to be shared: (i) 5€ (*n* = 177) mimicking a typical lab experiment, (ii) 100€ (*n* = 168), and (iii) 1,000€ (*n* = 171). The few papers on the role of stakes on giving behavior are quite inconclusive [6, 14]. The evidence ranges from strong negative effects of stakes [16] through mild [17–19] and no effects [20] to positive effects in [21]. Since the only study containing more than two stake levels is hypothetical [18] and the effects and experimental protocols differ across studies, one cannot make general inferences about how stakes affect generosity from the cited literature. Moreover, all the studies have been conducted under certainty regarding all aspects of the donation task. In contrast, the donations are incentivized and implemented in our experiments and we are interested in situations such as those listed above, in which ambiguity prevails.

In our environment, people commit to donate a fraction of a lottery prize before they learn whether they have won or not. Importantly, the subjects did not know the *precise* probabilities of winning (see Methods). As a result, people donate under ambiguity. Similarly to stakes, the evidence regarding how ambiguity affects giving is mixed. On the one hand, people may use uncertainty–be it risk or ambiguity–strategically to share less without affecting their social image, a phenomenon termed “moral wiggle room” [22]. [23] show that, in such contexts, selfishness appears more appropriate not only in the eyes of the decision-makers but also to others. [24–28] support this idea if the donated quantity is risky or ambiguous. Nevertheless, the effects are weaker and even disappear when the risk is either on the Dictators’ or both sides [25, 27]. On the other hand [29], document that uncertainty does not always promote selfishness. They contrast outcome uncertainty (uncertainty about whether the recipient is affected or not) and impact uncertainty (uncertainty about how much the recipient is affected), reporting that donations actually increase under impact uncertainty. Therefore, the evidence suggests that the extent of moral wiggling relies heavily on how uncertainty is introduced into the task. In particular, the asymmetry in the the impact of uncertainty across the donor and the recipient seems to be a key element for observing self-serving narratives in giving behavior.

Since we are mainly interested in the effect of stakes, the ambiguity is symmetric in our experiments in that both the kept and donated quantities are subject to the same degree of ambiguity. We expect such symmetric ambiguity to exert at most a small positive effect on donations because our manipulation makes the outcome uncertainty symmetric across both the donors and the recipients but leaves room for impact uncertainty as the donors cannot know how their donations affect the well-being of the recipients [29] and people cannot self-impose ignorance regarding the payment to both parties asymmetrically [30]. To assess the generalizability of our findings to certainty, we ran an additional treatment where people share 5€ under certainty. That is, they face a standard five-euro Dictator Game, in which all the payments were implemented with probability one (relegated to S1 File).

Since individual differences in generosity are essential for our understanding of the evolution and prevalence of cooperation [31] and another fundamental condition for extrapolating experimentally-observed behaviors to real life requires a certain degree of stability of behavior across contexts, we additionally ask whether the shares of a few particular behavioral types decrease, remain, or increase as we increase the pie to be split. Recent evidence documents that cooperative phenotypes are domain-general and temporally stable [32–34], suggesting that we may observe little variation in the number of people adhering to different altruistic types. We argue that the stakes at play are a fundamental element of the context and contribute to this literature by analyzing stakes within the same setting, as opposed to different settings under comparable stakes.

## Methods

We performed a series of experiments at the University Loyola Andalucía, Spain. Subjects were students enrolled in a series of courses across different fields of study and the two campuses of the University. A total of 539 students agreed to participate. The experimenters recruited the subjects for an experimental study to be conducted either in class or online (see below), in which they could earn money. Each student was only allowed to participate in one treatment. Students who participated first signed a written consent and then received the instructions explaining the anonymity rules, the procedures, and compensation in the experiment. Ethics Committee of the Universidad Loyola Andalucía approved the experiment and all participants signed an informed consent.

We performed three main treatments. In each treatment, subjects were asked–among other tasks–to donate to a charity of their choice or to an unknown charity (and not to other students) any fraction of a prize to be won with unknown probabilities. Since the experiment was performed in a jesuist University, the vast majority of subjects in the 5€ and 1,000€ treatments chose to donate to the jesuist non-governmental organization *Entreculturas*; other charities were also selected but rarely. All these donations were executed following the preferences of the subjects. For the sake of simplicity, all the donations in the 100€ treatment–that was conducted later–were made to an “unknown” charity and we transfered all the donations to *Entreculturas*.

The difference across the three main treatments was the prize to be split: 5€, 100€, or 1,000€. All subjects were informed that, during the experiment, they would have a chance to participate in a lottery, in which they can earn a fixed amount of money that varied across the treatments and that was known to the subjects while making their donations. However, no subject was informed about the likelihoods of earning these quantities etc. That is, they were entirely agnostic regarding the odds of earning the money. In the terminology of this study, the probabilities of earning the 5€, 100€, or 1,000€ were *ambiguous* for the participants.

The sample sizes were predefined by statistical power. The sample is large enough to detect an average effect of 0.3SD, with a power of 0.8 and a significance level of 95%. A total of 177 students participated in the treatment 5A (5€ under ambiguity) (mean age = 19.89, *SD* = 1.41) of which 61.71% were females; 168 students participated in the 100A treatment (mean age = 21.74, SD = 2.61) of which 56.54% were females; 171 students participated in the 1,000€ treatment (mean age = 19.70, *SD* = 0.99) of which 64.70% were females. Although the *gender* composition is somehow imbalanced across treatments, the percentage of women does not statistically differ across the three treatments (*p* > 0.31). For *Age* we find that the 100A sample is 1.8 year older than 5A (*p* = 0.000) but no different than 1000A (*p* > 0.130). Importantly, we do not find any effect of age nor gender on behavior (see also S1 File).

In all treatments, subjects were asked to donate a fraction of the prize from 0% to 100% in 10% increments. Subjects were fully aware of the commitment of their decisions and that they were not allowed to change their choices after the resolution of the lottery.

There are two important elements of the design to consider. First, treatments 5A and 1,000A were conducted at the University premises in classrooms, while the 100A treatment was run online due to the Covid-19 confinement during the very first week of the lock-down in Spain. Note that the 100€ treatment, an intermediate step between the 5€ and 1,000€ treatments, was planned to be conducted later but the pandemic situation obliged us to run it online. Recent evidence suggests that the online data are valid and comparable to those gathered in the lab [35–37]. The fact that the experiment was conducted during the confinement might have an impact on the donations though, but [38] show that University students do not change their giving behavior during the confinement. In [38], we show that behavior in the DG changes during the first days of the home confinment in March 2020 in southern Spain but this effect is only observed for non-student population above a certain age, while no behavioral shift is documented for young adult students who are comparable to the subject pool analyzed in the 5€ and 1,000€ treatments. Second, subjects actually made two or three donation decisions in the 5A and 1000A treatments, whereas they only made one decision in the 100A treatment. To provide a clear comparison across treatments, we only use the first donation for each subject.

## Results

**Fig 1:**
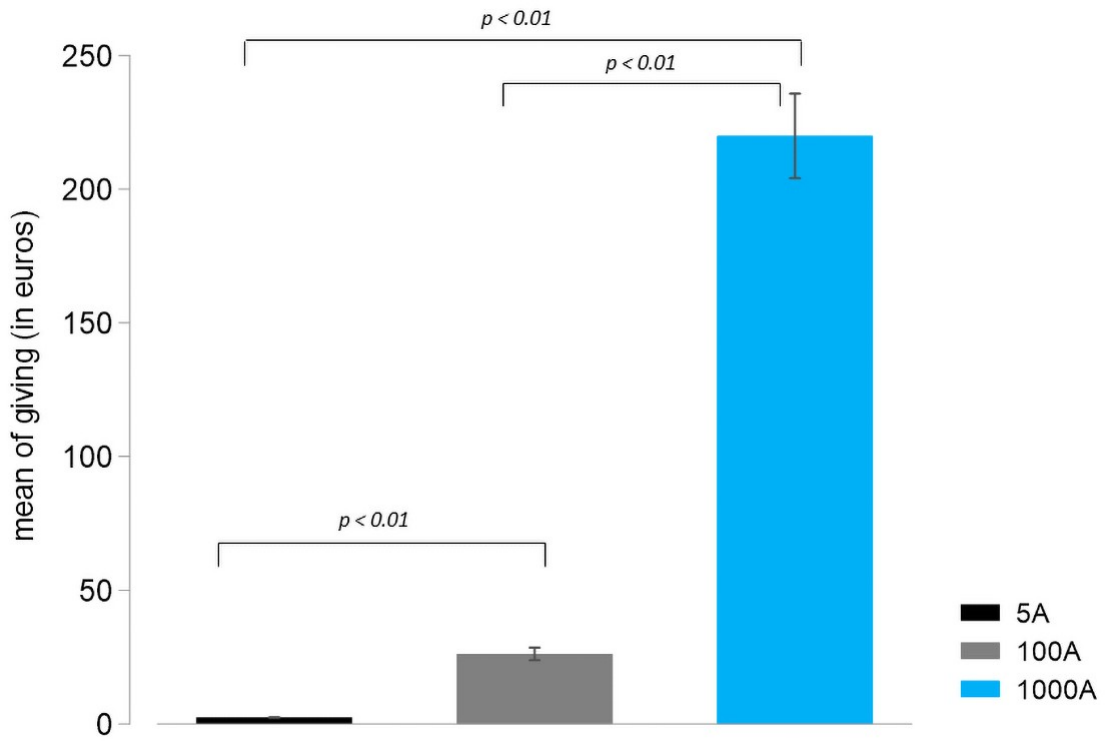
Average giving in the 5€, 100€, and 1000€ treatments. Grey vertical lines represent standard errors of the mean.

**Fig 2:**
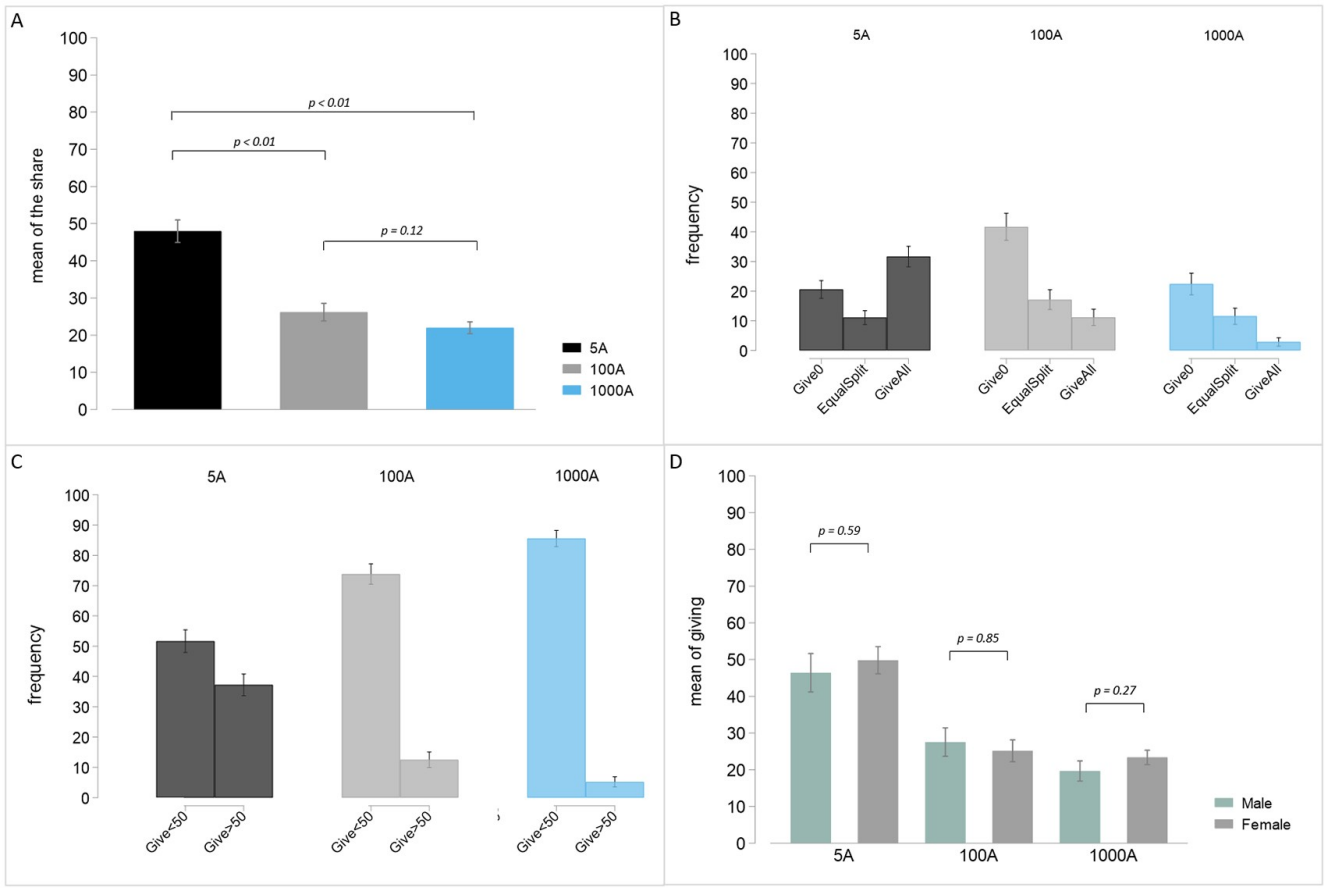
Additional behavioral patterns in the 5€, 100€, and 1000€ treatments. A. Average donated share of the pie. B. Shares of particular altruistic types: *Give*0 correspond to people giving nothing; *EqualSplit* correspond to people giving one half of the pie; *GiveAll* are people who donate all the money. C. Fractions of subjects donating less than 50% (*Give* < 50) or more than 50% (*Give* > 50). D. Average giving by gender. Grey vertical lines represent standard errors of the mean.

**Fig 3:**
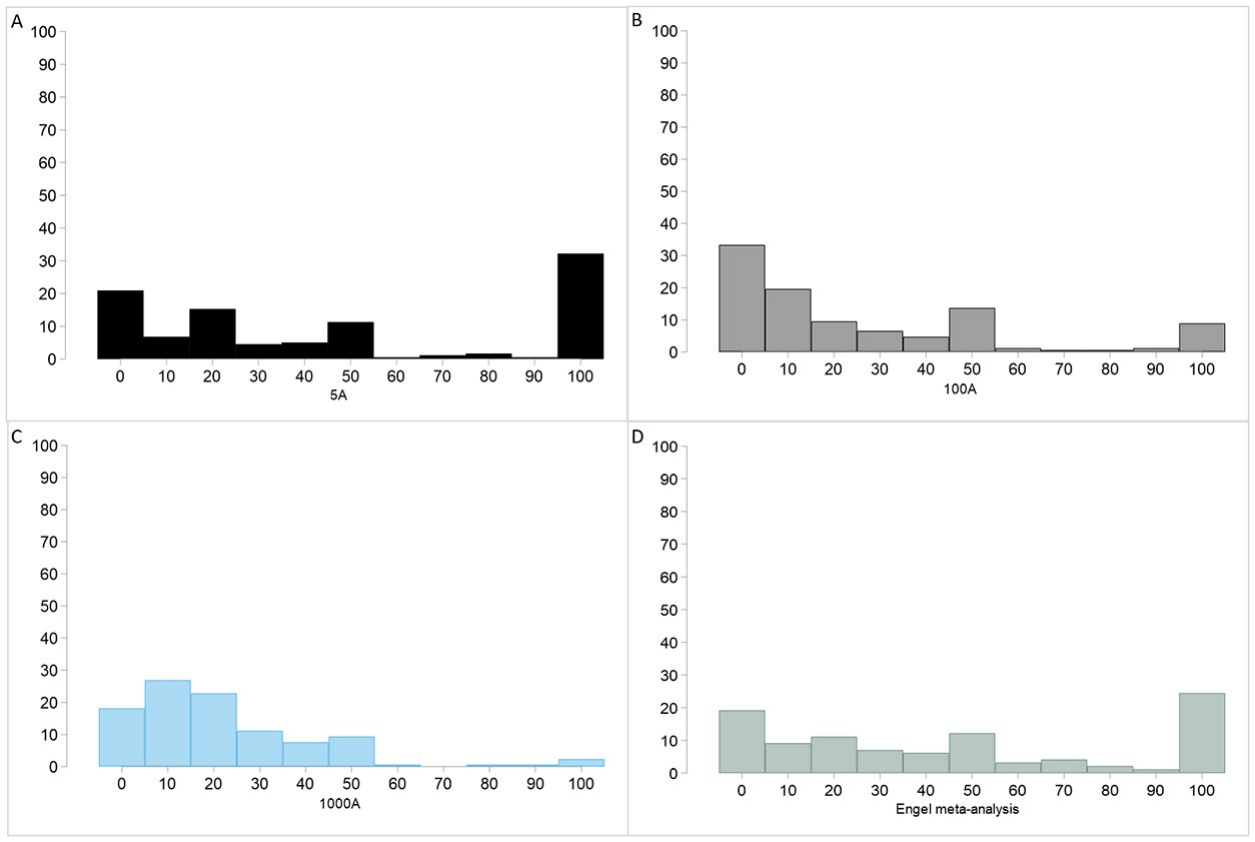
Distribution of donated shares across treatments (%). A. 5€. B. 100€. C. 1,000€. D. Donations to deserving recipients in Engel’s meta-study [6].

Fig 1 plots the average donation across treatments. In addition, Fig 2A plots the average donated fraction of the pie in each treatment; Fig 2B and 2C focus on particular behavioral types; Fig 2D plots the average donated share disaggregated by gender. Fig 3 displays the entire distributions of relative giving in our three treatments (panels A, B and C) and reproduces the distribution of donations to deserving recipients from Engel’s meta-analysis (panel D) [6]. To provide an alternative comparison across stakes, Fig 4 in S1 File provides the cumulative distributions of donated shares in each treatment.

Fig 1 shows that people give on average 2.40€, 26.19€, and 219.88€ (out of 5, 100, and 1000, respectively). All these differences are statistically significant (5A vs 100A: *p* = 0.000; 5A vs 1000A: *p* = 0.000; 100A vs 1000A: *p* = 0.000). That is, the absolute quantity donated increases dramatically across stakes. However, since the donations increase *sublinearly* with stakes, the average donated share of the pie decreases, as illustrated in Fig 2A. More precisely, subjects donate an average of 47.96% of the pie while sharing 5€. Nevertheless, the average fraction decreases significantly for 100€ (26.19%; t-test: *p* < 0.0001) and 1,000€ (21.98%: t-test: *p* < 0.0001). These differences are clearly visible in the distributions of donated shares in Fig 3, where the distributions in panels B and C (100 and 1,000€ treatments) are shifted to the left as compared to panels A and D. Although people give relatively less out of 1,000€ than 100€, the difference is not significant at conventional 5% (4.356; t-test: *p* = 0.124). We shall see below that this difference becomes significant once we control for age and gender. Hence, higher stakes induce people to donate more to charities but the donated share decreases.

The effect of stakes notwithstanding, subjects still donate an average of 20% of the prize and only about 20% donate zero even in the 1,000€ treatment, suggesting that only a minority behaves selfishly even for stakes as high as the average monthly income of people aged below 25 in Spain [39].

In Fig 2B, we concentrate on three particular behavioural types for their relevance in the literature: *selfish* individuals who donate zero, people who *share equally*, and *hyper-altruistic* subjects who give the whole pie to the charity. The only non-monotonic effect that we observe in the data is the fraction of entirely selfish people (labeled as *Give*0 in Fig 2B). We document 20.90% of selfish subjects under 5€ and 18.12% under 1,000€ (being both figures similar to Engel’s [6] meta-analysis, while this fraction increases to 34.94% for 100€. Whereas the difference between 5€ and 1000€ is not significant (t-test: *p* = 0.52), those between 5€ and 100€ and between 100€ and 1,000€ are significant at 1% (t-tests: *p* < 0.01). We have no explanation for this non-monotonicity but Fig 3 reveals that, if we compare the distributions in the 100€ and 1,000€ treatments, many people give zero or the minimum 10% in the former while the distribution is more concentrated on 10% and 20% of the pie in the latter. In fact, giving 0 or 10% are the most frequent gifts under 100€ (34.95% and 18.28%, respectively) in contrast to giving 10% and 20% (26.90% and 18.27%, respectively) under 1,000€. We would recover the monotonicity if we aggregate the fractions of people giving less than 30% in the three treatments (not reported, but see Fig 2C for a similar comparison).

The fraction of people sharing the pie equally (labeled as *Equal Split*) is largely stable across treatments (t-test: *p* > 0.40), around 10% of the sample regardless the stakes (see Fig 2B).

In sharp contrast, the share of people who donate the whole amount (*GiveAll*) decreases dramatically with stakes. Their fraction is similar for 5€ as compared to [6]. However the percentage of such *hyper-altruistic* subjects decreases significantly under both 100€ and 1,000€. The differences with respect 5€ are statistically strong (t-test: *p* < 0.001) as is the difference between 100€ and 1000€ (t-test: *p* = 0.008). In fact, such hyper-generosity virtually disappears under 1,000€: the percentage of subjects who donate the whole amount is only 2% and this fraction is statistically indistinguishable from zero (t-test: *p* = 0.181).

To provide an alternative look at subjects’ types, we classify people according to whether they give less or more than 50% in Fig 2C. The fraction of subjects giving less than 50% (*Give* < 50) monotonically increases from 50% to 86% with the stakes (proportion tests: *p* < 0.001 in all comparisons), whereas donating more than 50% (*Give* > 50) decreases steadily from 37% to 4% (proportion tests: *p* < 0.001). These figures suggest that not only giving the whole amount but also giving more than 50% is an artifact of the low stakes prevalent in the experimental literature.

Since there has been some debate regarding whether women are more generous than men [40–42] and whether each gender reacts differently to treatment manipulations [43], Fig 2D disaggregates the behavior by gender. We find neither gender differences within treatments nor differential treatment effects (t-tests: *p* > 0.1 for all comparisons). S1 File shows that these conclusions are robust to employing regression analysis.

Table 1 provides a more rigorous statistical analysis of the findings using ordinary least squares (OLS) regressions. Since the donations are bounded and the donated shares bounded and discrete percentages in the experiment, Table 3 in S1 File additionally delivers the corresponding tobit regression for the donated quantity and both the tobit and ordered logit regressions for the donated shares; the same table also provides probit estimations for the models in columns (3)–(7) in Table 1 where the dependent variables are binary. Since the results are robust to the estimated model, we focus on the OLS regression from Table 1 in the main text. All the regressions in Table 1 (as well as Table 3 in S1 File) have the same structure: we regress a variable of interest on the treatment dummies and control for age and gender; the baseline is the 5€ treatment in all cases.

**Table 1:** OLS estimation of the treatments effects.

	(1) *Giving*	(2) *Share*	(3) *Give*0	(4) *EqualSplit*	(5) *GiveAll*	(6) *Give* < 50	(7) *Give* > 50
100€	31.024***	-18.955***	0.111**	0.024	-0.225***	0.180***	-0.204***
	(5.430)	(4.559)	(0.053)	(0.041)	(0.048)	(0.060)	(0.053)
1000€	217.194***	-26.815***	-0.014	-0.021	-0.303***	0.348***	-0.327***
	(15.930)	(3.438)	(0.042)	(0.033)	(0.037)	(0.046)	(0.040)
female	9.831	0.922	-0.073*	0.027	-0.021	0.004	-0.031
	(11.094)	(2.983)	(0.040)	(0.028)	(0.031)	(0.039)	(0.033)
age	-3.633	-1.787	0.010	0.000	-0.007	0.020	-0.020
	(2.562)	(1.149)	(0.014)	(0.010)	(0.011)	(0.015)	(0.013)
Constant	68.601***	83.486***	0.045	0.095	0.470**	0.112	0.792***
	(52.640)	(23.320)	(0.281)	(0.193)	(0.229)	(0.300)	(0.273)
Observations	513	513	513	513	513	513	513
R-squared	0.397	0.125	0.034	0.005	0.136	0.102	0.136
F test model	69.933	16.012	4.197	0.602	17.110	14.651	17.572

Note: *Giving* is the amount (in euros) and *share* is the percentage of the pie given in the Dictator Game (DG). *Give*0 = 1 if a subject gives 0 in the DG and 0 otherwise. *EqualSplit*, *GiveAll*, *Give* < 50 and *Give* > 50 are defined in the same way. Robust standard errors in parentheses.

*** *p* < 0.01,

** *p* < 0.05,

* *p* < 0.1.

Columns (1–2) corroborate that people donate more in absolute terms but less in relative terms in both the 100€ and 1,000€ treatments, as compared to the 5€ case (*p* < 0.0001 in all cases). In addition, the estimated coefficient of the donated share in the 1,000€ treatment is 41% lower than under 100€, a difference significant at conventional 5% (Wald test, *p* = 0.04) for relative giving and strongly significant (*p* = 0.000) for absolute giving. Hence, pooling all the data from all treatments into one model and controlling for treatment differences, age, and gender simultaneously reveals that the sharing behavior is also different between the 100€ and 1,000€ treatments.

Column (3) analyzes how the proportion of selfish individuals differs across treatments. In line with the above findings, only the 100€ treatment generates more entirely selfish individuals (*p* = 0.04). As for people sharing the pie equally, column (4) corroborates that they do not differ across treatments. In contrast, column (5) confirms that higher stakes reduce the proportion of hyper-altruistic subjects compared to the 5€ treatment (*p* < 0.0001 in both cases) and between the 100€ and 1000€ treatments (Wald test: *p* < 0.0001). Finally, columns (6) and (7) show that the effect of stakes on the fraction of people giving less than 50% (*Give* < 50) and highly generous subjects (*Give* > 50), respectively, is in line with the findings discussed above: higher stakes lead to lower donated share. The fact that the sums of the treatment coefficients between columns (6) and (7) are statistically indistinguishable from zero (Chow tests, *p* > 0.50) further *suggests* that equal sharing is stable across treatments and that people who share more than 50% under low stakes *might potentially* switch to giving less than 50% under high stakes. Our between-subject design does not allow to test this statement formally though. All the reported results are robust to interacting the treatment effects with a female dummy (see Table 2 in S1 File).

To test the generalizability of our findings to certainty, we ran an additional treatment, in which people share 5€ under certainty. That is, they face a standard five-euro Dictator Game and their decisions were implemented with probability one. As hypothesized above, we find a mild positive effect of symmetric ambiguity on giving as compared to such control treatment. More precisely, subjects donations are reduced by 11.46% under certainty (*t* − *test* = 2.04, *p* = 0.043; see S1 File for more details). Since the difference is only significant at 5%, we make two conclusions. First, our results contrast the unconditional claims that people exploit uncertainty to hide their selfishness and one of our contributions is to point out that the effect of uncertainty on moral wiggling is limited: moral wiggles disappear under the symmetric ambiguity employed in our study. Second, the small difference between the certainty and symmetric ambiguity makes us believe that the documented role of stakes might be generalized to certainty under the assumption that stakes affect donations equally under both certainty and symmetric ambiguity. Future research should answer whether this assumption holds.

## Conclusion

Using an incentivized experiment with statistical power, this paper analyzes how stakes affect giving to charity. We show that, although the donations increases absolutely, the donated shares are dramatically reduced as the stakes increase. Hence, our results support the findings of [16] but contrast those of mild, null, or positive effects documented elsewhere [17–21]. It is worth stressing that the two common features of both [16] and our study is the employment of stakes as high as monthly salaries. The difference is that their experimental subjects were from Bangladesh, a considerably poor society, while ours come from a developed country.

As for different behavioral types, we show that fair behavior–namely, sharing equally–is remarkably stable across stakes. In contrast, both giving more than 50% and giving the whole amount to charity virtually vanish as we increase the stakes. Hence, such hyper-altruistic behavior observed in the experimental literature seems to be an artifact of low stakes typically employed in psychological and economic experiments.

Our findings have implications for scholars, policy-makers, and fundraisers. As for the scientific contribution, we first show that sharing with others is a prevalent aspect of human nature because people still share considerably (over 20%) in our experiment even for stakes as high as an average monthly salary of young people at the same age as our experimental subjects [39].

On the other hand, since the degree of generosity is highly sensitive in both absolute and relative terms on stakes, our second message is that theoretical modeling and simulating of models of human altruism and cooperation should carefully account for how these phenomena depend on the stakes of the modeled environment. We suggest that modeling generosity in, say, typically day-to-day interactions may be subject to a higher degree of prosociality than behavior of brokers in the stock market, where the stakes at play are considerably higher. Data as ours might provide a guidance in this respect.

Third, we contradict the conclusions that cooperative and behavioral types are universally stable. The literature has focused on different strategic settings [32–34], while we analyze varying stakes within the same setting. Nevertheless, stakes are a fundamental aspect of the context. As a result, the definition of the different behavioral phenotypes should account for how each type depends on the underlying incentives, as already suggested elsewhere (e.g. [14]).

Last, we only partially understand the underlying mechanisms behind the excuse-driven behavior under uncertainty [22, 30]. Do people exploit uncertainty as an excuse to share less with other generally or are there limits to such moral wiggling? Are moral wiggles under uncertainty a moral or cognitive phenomenon? Since people give slightly more under symmetric uncertainty as compared to certainty in our experiment, our data suggest that the role of uncertainty as a source of moral wiggling is limited and relies on the asymmetry across the donor and the recipient. If we remove such asymmetry, only impact uncertain remains and we reproduce the results of [29]. Hence, the role of uncertainty as a trigger of moral wiggles is not unlimited.

Policy-makers appealing to concerns toward others should predict correctly how their recommendations depend on the contextual incentives. For example, many anti-Covid-19 policies, such as mask-wearing or social distancing, appeal strongly to the effects of one’s behavior on others. Our data suggest that these policies might be ineffective if the policy-makers estimate the effects based on social concerns measured under standard laboratory experiments. These considerations naturally apply to other policies such as the enhancement of environmentally friendly behaviors, charity giving, and so forth.

Regarding fundraising, our study informs the design of certain fundraising campaigns. For instance, if one has a fixed amount of money to distribute via ruffles to the public and expects a share to be donated to charities, more money would be collected if there were more small prizes for many people than one unique high prize for one winner. If, in contrast, the fundraiser can choose whether to raise funds from the same number of people of differing wealth conditions, our results show that more money would be raised on average from richer people.

## Supporting information

S1 File (PDF)

S1 Data (CSV)
