# Supplementary material for: Hyper-altruistic behavior vanishes with high stakes
Source: PLoS One. 2021 Aug 25;16(8):e0255668. doi: 10.1371/journal.pone.0255668 (PMC8386826; doi:10.1371/journal.pone.0255668)
Supplement: S1 File — (PDF) [file pone.0255668.s001.pdf]

S.1 Additional Information

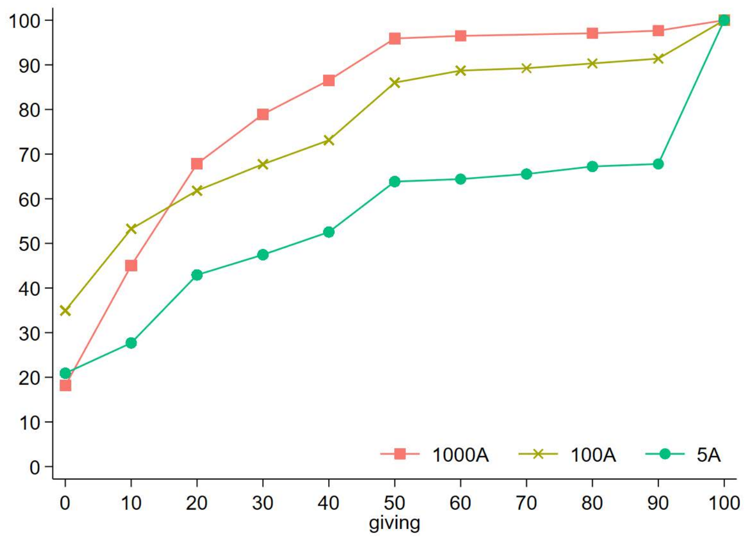

Fig 4. Cumulative Frequencies of Donations across Treatments.

S.2 Sharing 5€ under certainty

Apart from the main treatments in the main text, we additionally conducted a treatment, in which people donated out of 5€ to be received with certainty. That is, people played a standard five-euro Dictator Game. The sample in this treatment was also powered to detect an average effect of 0.33SD with a power of 0.8 and a significance level of 90% while comparing this treatment with the 5A conditions. A total of $n = 104$ students participated in this treatment under conditions identical to our main treatments (see *Methods*). We compare the donations in this treatment with the behavior in the 5A treatment from the main text. Below, we label $DG5$ the treatment under certainty and $DG5A$ the 5A treatment.

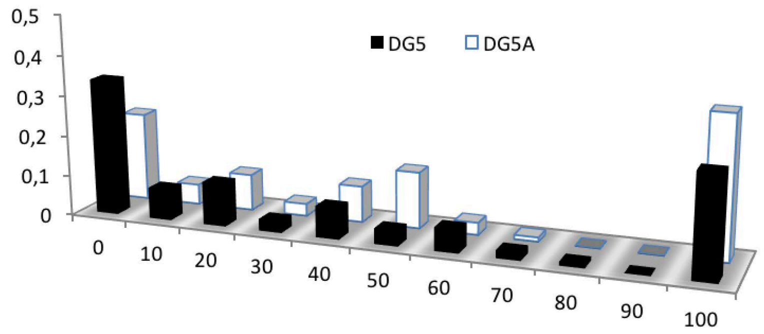

Fig 5. Donations in DG5 and DG5A

Figure 5 compares the distributions of donations in the two treatments. Both distributions are very similar to the distributions reported in the literature on the DG against a charity, including the modal choice of donating the whole pie (see Figure 3D in the main text or Figure 4 in [6]. Figure 5 suggests that people give somewhat more in $DG5A$ than in $DG5$. There seems to be more subjects keeping the entire endowment for themselves and fewer people sharing around 50% and 100% of the pie in the latter case. Indeed, subjects donate 50.79% of the pie in $DG5A$ while the fraction drops to 39.32% in $DG5$. This difference of 11.46 percentage points is statistically significant at conventional 5% ($t - test = 2.04$, $p = 0.043$). We thus conclude that subjects do not use *symmetric* ambiguity as an excuse to give less in our experiment. In fact, they give more. Although we do not isolate the effect of impact uncertainty as proposed by [29], the symmetric ambiguity regarding the payment of both parties seems to eliminate moral wiggling and impact ambiguity seems to prevail, reproducing the result of [29].

S.3 Treatment effects disaggregated by gender

Table 2. Gender heterogeneous treatments effects.

	(1)<br>*Giving*	(2)<br>*Share*	(3)<br>*Give*0	(4)<br>*EqualSplit*	(5)<br>*GiveAll*	(6)<br>*Give* < 50	(7)<br>*Give* > 50
100€	32.254***	-15.744**	0.028	0.033	-0.207***	0.168*	-0.201**
	(6.228)	(7.102)	(0.082)	(0.054)	(0.074)	(0.087)	(0.081)
1000€	192.423***	-27.609***	-0.029	-0.006	-0.298***	0.371***	-0.365***
	(27.593)	(5.956)	(0.079)	(0.050)	(0.062)	(0.075)	(0.065)
female	-2.054	2.405	-0.130**	0.040	-0.008	0.008	-0.048
	(1.689)	(6.449)	(0.066)	(0.048)	(0.073)	(0.079)	(0.076)
100×female	-1.864	-5.469	0.142	-0.016	-0.031	0.022	-0.006
	(4.931)	(8.045)	(0.098)	(0.072)	(0.086)	(0.104)	(0.092)
1000×female	38.710	1.152	0.026	-0.024	-0.008	-0.035	0.058
	(33.911)	(7.288)	(0.093)	(0.067)	(0.078)	(0.095)	(0.082)
age	-4.057	-1.809	0.010	0.000	-0.007	0.021	-0.021
	(2.572)	(1.158)	(0.014)	(0.010)	(0.011)	(0.015)	(0.013)
Constant	84.389	83.009***	0.080	0.083	0.462**	0.102	0.815***
	(51.935)	(23.696)	(0.281)	(0.200)	(0.234)	(0.306)	(0.275)
Observations	513	513	513	513	513	513	513
R-squared	0.402	0.127	0.039	0.005	0.137	0.103	0.137
F test model	47.901	10.833	3.471	0.422	11.660	9.857	11.922

Note: *Giving* is the amount (in euros) and *share* is the percentage of the pie given in the Dictator Game (DG). *Give*0 is equal 1 if subject gives 0 in the DG, and 0 otherwise. *EnqualSplit*, *GiveAll*, *Give* < 50 and *Give* > 50 are defined in the same way. Robust standard errors in parentheses. *** $p < 0.01$, ** $p < 0.05$, * $p < 0.1$.

S.4 Robustness check

Table 3. Non-linear estimations of the treatments effects.

	(1) *Share* (order. logit)	(2) *Share* (tobit)	(3) *Give*0 (probit)	(4) *EqualSplit* (probit)	(5) *GiveAll* (probit)	(6) *Give* < 50 (probit)	(7) *Give* > 50 (probit)
100€	-0.978***	-18.955***	0.110**	0.023	-0.128***	0.150***	-0.132***
	(0.262)	(4.541)	(0.054)	(0.040)	(0.027)	(0.049)	(0.034)
1000€	-1.078***	-26.815***	-0.015	-0.022	-0.217***	0.309***	-0.249***
	(0.196)	(3.424)	(0.047)	(0.033)	(0.025)	(0.037)	(0.027)
female	0.179	0.922	-0.077*	0.027	-0.023	0.004	-0.029
	(0.173)	(2.972)	(0.040)	(0.028)	(0.029)	(0.042)	(0.033)
age	-0.103	-1.787	0.010	0.000	-0.007	0.021	-0.019
	(0.064)	(1.144)	(0.013)	(0.010)	(0.010)	(0.015)	(0.012)
Constant		83.486*** (23.229)					
Observations	513	513	513	513	513	513	513

Note: *Share* is the percentage of the pie given in the Dictator Game (DG). *Give*0 = 1 if a subject gives 0 in the DG and 0 otherwise. *EqualSplit*, *GiveAll*, *Give* < 50 and *Give* > 50 are defined in the same way. Robust standard errors in parentheses. *** $p < 0.01$, ** $p < 0.05$, * $p < 0.1$.

S.5 Experimental instructions

In the following sections, we present the instructions that faced subjects in each treatment. The experiment was conducted in Spain. In the following we show the translated instructions.

Note that in the 5€ and 1,000€ treatments, we let participants choose the charity that would receive their donations by writing it into a space in the instruction right below the selection of the donated fraction. Since the experiment was performed in a jesuist University, the vast majority of subjects chose to donate to the jesuist non-governmental organization *Entreculturas* (www.entreculturas.org), but other NGOs were also selected despite rarely. All the donations in these treatments were executed following the preferences of the subjects. To simplify the organization of the 100€ treatment, we let subjects only choose how much to donate to an unkown charity and we transfered all the donated money to *Entreculturas* too.

Treatment 5€

If you win the 5€ lottery, would you like to donate a fraction to an NGO? Keep in mind that the answer you give us is NOT hypothetical and will determine your final payment. Please mark the percentage of the prize that you would donate on the scale below.

Treatment 100€

If you win the 100€ lottery, would you like to donate a fraction to an NGO? Select the amount of money you want to donate from 0 (nothing) to 100% of 100 euros. Please mark only one percentage.

Treatment 1000€

If you win the 1000 euros lottery, would you like to donate a fraction to an NGO (see below)? Keep in mind that the answer you give us is NOT hypothetical and will determine your final payment. Please mark the percentage of the prize that you would donate on the scale below.
